# Imaging of inflammatory cellular protagonists in human atherosclerosis: a dual-isotope SPECT approach

**DOI:** 10.1007/s00259-020-04776-0

**Published:** 2020-04-15

**Authors:** Hilary E. Barrett, Eric J. Meester, Kim van Gaalen, Kim van der Heiden, Boudewijn J. Krenning, Freek J. Beekman, Erik de Blois, Jan de Swart, H J Verhagen, Theodosia Maina, Berthold A. Nock, Jeffrey P. Norenberg, Marion de Jong, Frank J. H. Gijsen, Monique R. Bernsen

**Affiliations:** 1 Biomedical Engineering, Department of Cardiology, Erasmus MC, Rotterdam, the Netherlands; 2 Department of Radiology & Nuclear Medicine, Erasmus MC, Rotterdam, the Netherlands; 3 Department of Cardiology, Erasmus MC, Rotterdam, the Netherlands; 4 Department of Vascular Surgery, Erasmus MC, Rotterdam, the Netherlands; 5 MiLabs, B.V., Utrecht, the Netherlands; 6 Section Biomedical Imaging, Delft University of Technology, Delft, the Netherlands; 7 Department of Translational Neuroscience, Brain Centre Rudolf Magnus, University Medical Centre Utrecht, Utrecht, the Netherlands; 8 Molecular Radiopharmacy, INRASTES, NCSR “Demokritos”, 15310 Athens, Greece; 9 Radiopharmaceutical Sciences, University of New Mexico, Albuquerque, NM USA; 10 Applied Molecular Imaging Erasmus Core Facility Erasmus MC, Rotterdam, the Netherlands

**Keywords:** Atherosclerosis, Inflammation, Carotid artery, Macrophage, Leukocyte, SPECT imaging, Dual-isotope

## Abstract

**Purpose:**

Atherosclerotic plaque development and progression signifies a complex inflammatory disease mediated by a multitude of proinflammatory leukocyte subsets. Using single photon emission computed tomography (SPECT) coupled with computed tomography (CT), this study tested a new dual-isotope acquisition protocol to assess each radiotracer’s capability to identify plaque phenotype and inflammation levels pertaining to leukocytes expressing leukocyte function-associated antigen-1 (LFA-1) and the leukocyte subset of proinflammatory macrophages expressing somatostatin receptor subtype-2 (SST_2_). Individual radiotracer uptake was quantified and the presence of corresponding immunohistological cell markers was assessed.

**Methods:**

Human symptomatic carotid plaque segments were obtained from endarterectomy. Segments were incubated in dual-isotope radiotracers [^111^In]In-DOTA-butylamino-NorBIRT ([^111^In]In-Danbirt) and [^99m^Tc]Tc-[N^0–1^_4_,Asp^0^,Tyr^3^]-octreotate ([^99m^Tc]Tc-Demotate 2) before scanning with SPECT/CT. Plaque phenotype was classified as pathological intimal thickening, fibrous cap atheroma or fibrocalcific using histology sections based on distinct morphological characteristics. Plaque segments were subsequently immuno-stained with LFA-1 and SST_2_ and quantified in terms of positive area fraction and compared against the corresponding SPECT images.

**Results:**

Focal uptake of co-localising dual-radiotracers identified the heterogeneous distribution of inflamed regions in the plaques which co-localised with positive immuno-stained regions of LFA-1 and SST_2_. [^111^In]In-Danbirt and [^99m^Tc]Tc-Demotate 2 uptake demonstrated a significant positive correlation (*r* = 0.651; *p* = 0.001). Fibrous cap atheroma plaque phenotype correlated with the highest [^111^In]In-Danbirt and [^99m^Tc]Tc-Demotate 2 uptake compared with fibrocalcific plaques and pathological intimal thickening phenotypes, in line with the immunohistological analyses.

**Conclusion:**

A dual-isotope acquisition protocol permits the imaging of multiple leukocyte subsets and the pro-inflammatory macrophages simultaneously in atherosclerotic plaque tissue. [^111^In]In-Danbirt may have added value for assessing the total inflammation levels in atherosclerotic plaques in addition to classifying plaque phenotype.

## Introduction

Atherosclerosis is derived from an inflammatory cycle that forms in response to the deposition of cholesterol-rich, apolipoprotein B-containing lipoproteins in the intimal layer of the arterial wall [1]. Inflammation controls all stages of the disease starting from the initiating atheroma development to the advanced, and potentially rupture-prone, plaque. Inflammation is instrumental in causing the clinical complications resulting in life-threatening stroke and myocardial infarction [2]. Detection of this inflammatory activity is a high clinical priority. If the atherosclerosis-associated inflammation can be detected early, a considerable reduction in the occurrence of these cardiovascular events could be made possible.

From the initiating phase of the disease, leukocytes, including monocyte-derived macrophages, lymphocytes and neutrophils, adhere to activated endothelium and are recruited into the developing atheroma [3]. The proinflammatory leukocytes elicit a cascade of biological processes which renders the plaque susceptible to structural disruptions including fibrous cap ruptures [4]. Following rupture, exposure of the thrombogenic plaque core to blood components can result in catastrophic cardiovascular events [5]. The search for methods that can specifically target the inflammatory activity in atherosclerotic tissue and identify the high-risk vulnerable plaque phenotype addresses a clinical need [6].

Molecular imaging techniques including positron emission tomography (PET) and single photon emission computed tomography (SPECT) have the unique capability to target specific biological processes at the molecular level. Numerous radiotracers, capable of targeting leukocytes and the inflammatory subsets, have been developed and imaged in human atherosclerotic plaque vessels [7]. [^111^In]In-DOTA-butylamino-NorBIRT also known as [^111^In]In-Danbirt is a novel radiotracer which can specifically target the leukocytes which express leukocyte function-associated antigen-1 (LFA-1). The value of this radiotracer has been initially demonstrated in in vivo animal studies utilising atherosclerosis disease models and also in a human atherosclerotic plaque case [8, 9]. Targeted imaging of atherosclerotic plaque with [^68^Ga]Ga-DOTA-[Tyr^3^]octreotate, a somatostatin receptor subtype 2 (SST_2_)–targeting radiotracer, detects the presence of activated (proinflammatory) macrophages [10]. The clinical utilisation of this radiotracer has revealed its ability to achieve more focal imaging of inflamed atherosclerotic plaques in comparison to 2-deoxy-2-[^18^F]fluoro-D-glucose ([^18^F]FDG) and thereby presenting another viable radiotracer [10–12]. Clinical studies indicated that the detection of proinflammatory macrophages with a SST_2_ targeting radiotracer could help discriminate culprit plaques which have an overall higher uptake [10, 11]. Notwithstanding, there are conflicting results reported for this radiotracer. For example, comparing symptomatic culprit vessels to the contralateral vessels in the carotid vasculature can result in little to no differences in terms of uptake levels [13, 14]. The detection of ‘culprit’ inflamed vessels is partially impeded by the paucity of information linking radiotracer uptake with respect to the specific plaque phenotype.

Furthermore, the simultaneous use of two radiotracers, targeting different molecular processes, with dual-isotope acquisition SPECT imaging has provided an enhancement in the understanding of specific diseases [15–17]. The utilisation of a dual-isotope acquisition protocol for atherosclerotic plaque tissue assessment may also provide improved information and in terms of aiding the detection of specific plaque phenotype. In this regard, this study aims to assess the added value of simultaneously targeting all leukocytes expressing LFA-1 and the subset of proinflammatory macrophages expressing SST_2_ in order to identify plaque phenotype. To investigate this, a dual-isotope acquisition protocol of [^111^In]In-Danbirt and [^99m^Tc]Tc-[Asp,Tyr]-octreotate also known as [^99m^Tc]Tc-Demotate 2 [18] is utilised to image ex vivo human carotid artery plaques. Additionally, the two radiotracers are compared to investigate if targeting all leukocytes provides pertinent information regarding the inflammatory status of the plaque that could be overlooked with current clinical protocols targeting the subset of proinflammatory macrophages alone.

## Materials and methods

### Study design

A cross-sectional molecular imaging study was performed with excised human carotid plaques. This exploratory research examines the uptake of two radiotracers pertaining to inflammatory populations involved in plaque progression. The plaque samples were obtained from seven atherosclerotic/symptomatic patients through endarterectomy procedures in the Erasmus MC, Rotterdam, The Netherlands, in a manner that conformed to the declaration of Helsinki and was approved by the hospital’s ethical research committee (MEC 2008-147). The carotid plaques were divided into segments of 2-mm thickness (*n* = 23) for SPECT/CT imaging followed by immunohistochemistry. In another set of experiments, adjacent carotid endarterectomy (CEA) segments (*n* = 7) were sectioned for autoradiography followed by immunohistochemistry.

### Radiolabelling

**Fig. 1:**
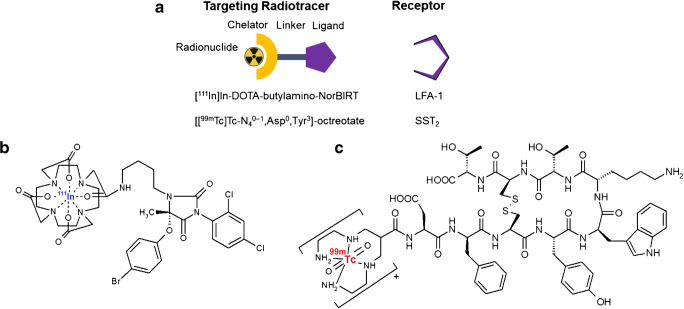
**a** Schematic of the two radiotracers targeting the receptors of leukocytes expressing leukocyte function-associated antigen-1 (LFA-1) and proinflammatory macrophages expressing somatostatin receptor subtype-2 (SST_2_). Chemical structures of the two radiotracers (**b**) [^111^In][In-DOTA-butylamino-NorBIRT] ([^111^In]In-Danbirt) and (**c**) [[^99m^Tc]Tc-[N^0–1^_4_,Asp^0^,Tyr^3^]octreotate ([^99m^Tc]TcTc-Demotate 2)

**Fig. 2:**
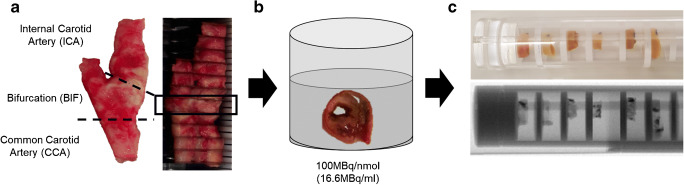
**a** Carotid plaque sample sectioned into 2-mm segments, (**b**) incubation step involved immersing segment in mixture of dual-radiotracers [^111^In]In-Danbirt) and [^99m^Tc]Tc-Demotate 2 and (**c**) live and x-ray view of plaque segments (2 mm) mounted on a custom-made holder for dual-isotope acquisition SPECT/CT scanning

To target the two plaque’s inflammatory cell populations, [^111^In]In-Danbirt (MW = 886.5 g/mol) [9] and [^99m^Tc]Tc-Demotate 2 [18] were used (Fig. 1). Each peptide was radiolabelled with a molar activity of 100 MBq/nmol, the radiochemical purity was > 90%, and the incorporation yield was > 99%, as determined by high-pressure liquid chromatography and instant thin-layer chromatography on silica gel. For [^111^In]In-Danbirt Quenchers (3.5 mM ascorbic acid, 3.5 mM gentisic acid, 10 mM methionine) were present during labelling to prevent radiolysis of the radiotracer [19]. The radiotracers were mixed in a solution of phosphate-buffered saline (PBS) containing 0.1% v/v bovine serum albumin (BSA). The plaque samples were incubated in a mixed solution of the 2 radiotracers for 1 h at room temperature. The plaque samples were then washed in PBS five times and assembled on a sealed Perspex sample holder for scanning (Fig. 2).

### Dual-isotope SPECT/CT imaging

For SPECT/CT imaging a VECTor5/CT system (MILabs B.V. Utrecht, The Netherlands) was used equipped with a high-energy ultra-high resolution mouse (HE-UHR-M) collimator. The system equipped with this collimator yields a 0.5 mm reconstructed SPECT resolution and a SPECT sensitivity of ~ 0.3% [20, 21]. Each scan was acquired in list mode using the same scan parameters, spiral scan mode [22], and fine step mode at 30 s per bed position yielding a total acquisition time of 90 min for a field of view of 26 mm in diameter and 48 mm in length. This permitted scanning six 2-mm plaque segments, placed in the sample holder, during a single scan. For the CT, scanner settings were identical for all scans. Ultra-focus magnification was applied with a full scan angle, at 0.24 mA, 50 kV and 75 ms yielding a total scan time of 15 min. The scans were reconstructed using filtered back projection at a resolution of 20 μm and downsampled to a reconstructed resolution of 80 μm for registration to SPECT scans.

To accomplish quantification of ^99m^Tc and ^111^In from dual isotope acquisitions, two separate reconstructions were performed. For ^99m^Tc, a width of 20% of the gamma photopeak at 140 keV was incorporated in the reconstruction. For ^111^In, two photopeak windows were set incorporating a width of 20% of the each gamma photopeak at 171 and 245 keV [23]. For scatter correction the triple-energy-window method was [24] applied to each photo peak. Phantom experiments with dual-isotope acquisitions for ^99m^Tc and ^111^In were performed using the small-animal SPECT phantom SPECT IQ phantom which verified that the cross-talk within the relevant energy ranges was negligible [25].

SPECT scans were reconstructed at 0.2-mm voxel size using the similarity regulated ordered subset expectation maximisation (SROSEM) algorithm [22]. For this, 9 iterations, 128 subsets and a 3D Gaussian post filter of 0.5 mm (FWHM) were utilised. The VECTor5/CT system was calibrated to a standard of known activity that was measured in a dose calibrator, for absolute SPECT quantification for both radionuclides. All analysis of SPECT/CT scans was performed using PMOD (PMOD Technologies LLC, Zürich, Switzerland, Version 3.4). After imaging, the tissue segments were embedded in Tissue-Tek medium and stored at − 80 °C for immunohistological analysis after the radioactivity contained in the samples had decayed.

### Immunohistological analyses

Each 2-mm plaque segment was cryo-sectioned into four 500-μm blocks in line with the SPECT resolution. Within each 500-μm block, sequential adjacent sections of 5 μm were made which overall yielded a total of 262 sections for quantification and analysis with respect to the SPECT/CT scan data. Sections were immunohistochemically stained with anti-LFA-1 (mouse anti-human CD11a 1:100 Biorad, MCA1848 clone 38), and anti-SST_2A_ (human tissue: rabbit anti-human SSTR2A 1:50, clone UMB1, Abcam). Haematoxylin and eosin staining was used to determine the plaques overall structural morphology. The slides were photographed using a NanoZoomer Digital slide scanner (Hamamatsu, Photonics K.K). All images were exported to BioPix iQ 3.3.5 software for quantitative analysis. The areas of positively stained LFA-1 and SST_2_ were quantified using the dedicated hue, saturation and brightness selection tool, and positive areas were measured as a percent fraction of the total plaque tissue area.

### Plaque classification

The twenty-three carotid plaque segments were classified into 3 groups according to the American Heart Association (AHA) plaque classification [4], based on distinct morphological characteristics, identified with histology. Segments with pathological intimal thickening (*n* = 4, 17%) contained areas of inflammatory infiltration without necrotic tissue and were present in the bifurcation and internal carotid region. The fibrous cap atheroma segments (*n* = 9, 39%) morphologically consisted of a well-formed necrotic core and an overlying fibrous cap containing a high infiltration of inflammatory cells and were mainly found in the internal and common carotid regions. The fibrocalcific segments (*n* = 10, 43%) contained large areas of calcification within the necrotic core and were all located in the bifurcation region.

### Autoradiography

Segments of the carotid plaques, adjacent to those used for scanning, were cryo-sectioned at 10 μm and examined by ex vivo autoradiography and immunohistochemistry. Slides were washed in a washing buffer consisting of 1 M Tris-HCl (pH = 7.6) containing 0.25% v/v BSA for 10 min. The samples were incubated with 80 μL of radiotracer (10^−9^ M), with and without excess (10^−6^ M) matching unlabelled tracer for blocking. Slides were then washed and allowed to dry in an incubator at 37 °C. High sensitivity phosphor screens were placed over the tissue sections for 48 h, after which the screens were read using a Packard Cyclone (Perkin Elmer) resolution 600 DPI. Quantification of the plaque sections was performed in terms of digital light units per mm^2^ (DLU/mm^2^) using Optiquant (Perkin Elmer).

### Statistical analysis

Statistical analysis was performed using IBM SPSS statistics 21. Shapiro–Wilk tests were performed to assess the distribution of the data and select the most appropriate statistical test. Pearson’s correlation (*r*) and Spearman’s rho correlation (*r*_s_) were used to compare the correlation between the uptake levels of the radiotracers. A Mann–Whitney *U* test was used for non-parametric datasets to compare differences between uptake and positive area fraction for the three plaque morphology groups.

## Results

### Expression of LFA-1 and SST_2_ in carotid plaque segments

**Fig. 3:**
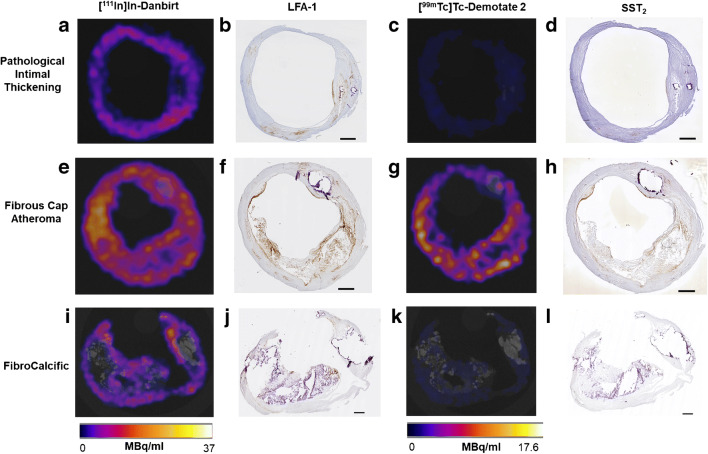
SPECT images of uptake for [^111^In]In-Danbirt (left panel 0–37 MBq/ml) and [^99m^Tc]Tc-Demotate 2 (right panel 0–17.5 MBq/ml) and the corresponding immunohistological sections classified according to plaque phenotype; pathological intimal thickening (**a**–**d**), fibrous cap atheroma (**e**–**h**) and fibrocalcific (i–l) (scale bar = 1 mm)

**Fig. 4:**
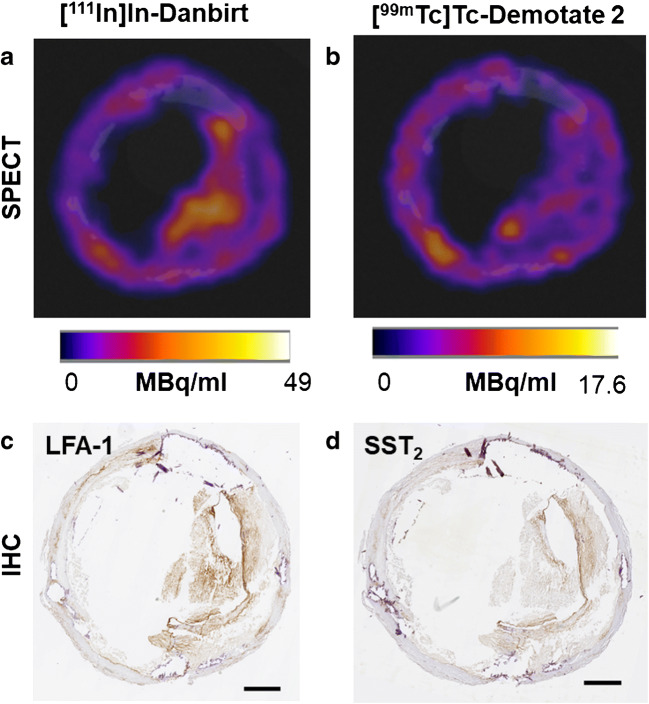
SPECT co-localisation of radiotracer uptake (**a**) [^111^In]In-Danbirt) and (**b**) [^99m^Tc]Tc-Demotate 2 and corresponding immunohistochemistry sections with positive immunostaining for (**c**) LFA-1 and (**d**) SST_2_ in a plaque segment classified with fibrous cap atheroma phenotype (scale bar = 1 mm)

**Fig. 5:**
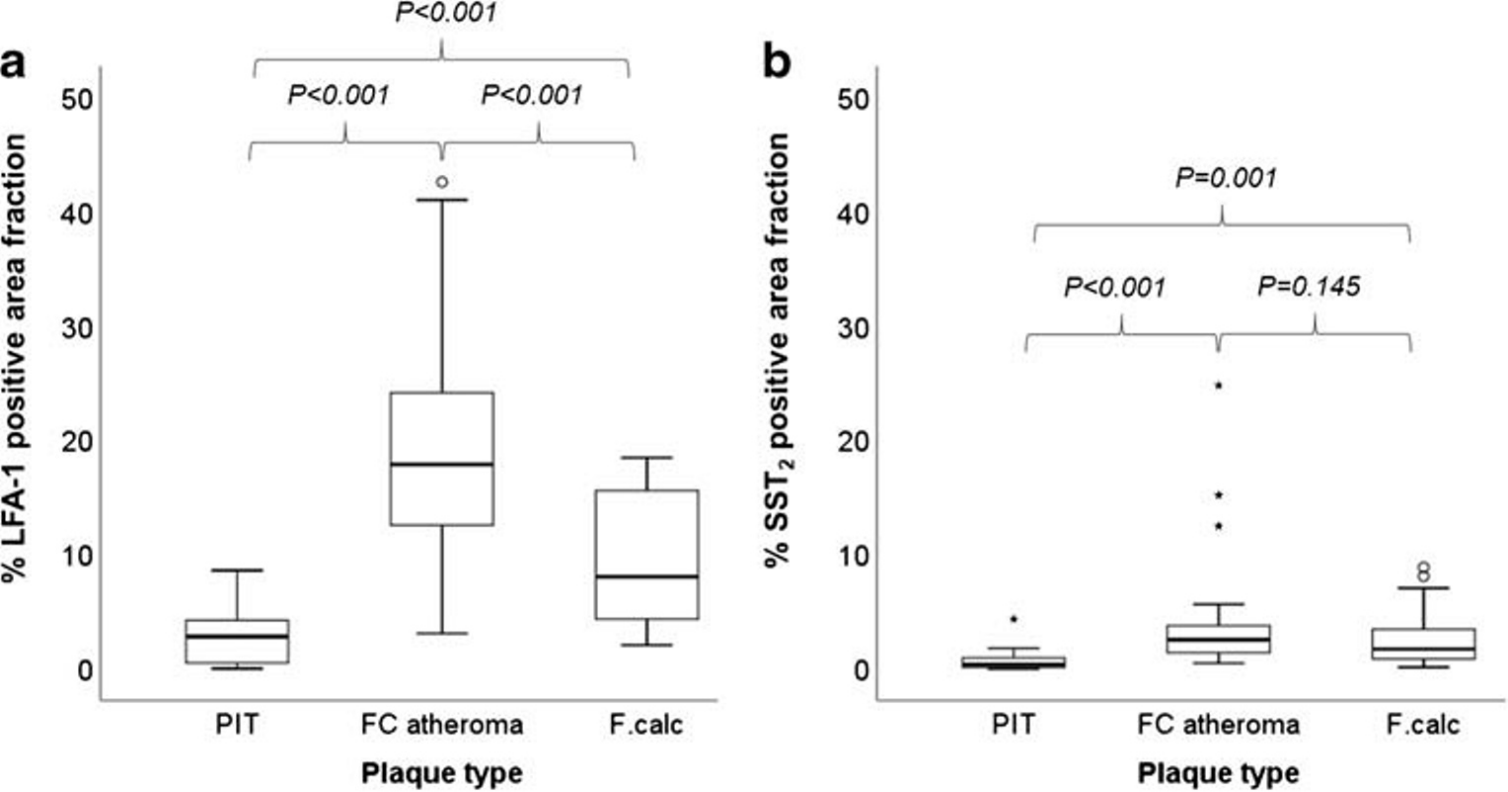
Box-plot of percent area fraction of positive immuno-staining for (a) LFA-1 and (b) SST_2_ in segments classified according to plaque phenotype (PIT, pathological intimal thickening; FC atheroma, fibrous cap atheroma; F.calc, fibrocalcific)

Figure 3 illustrates the distinct morphological characteristics and positive immuno-stained regions of LFA-1 and SST_2_ in three plaque phenotypes classified as pathological intimal thickening, fibrous cap atheroma and fibrocalcific. In the inflamed fibrous cap atheroma phenotype, immunohistological analyses revealed large clusters of co-localising LFA-1- and SST_2_-expressing inflammatory cells concentrated in the atheroma, at the fibrous caps and along shoulders (Figs. 3f, h and 4c, d). Quantification of the areas with positive immuno-staining for LFA-1 and SST_2_, within the representative 4 cryosections taken at intervals of 500 μm within each of the 2-mm plaque segments, revealed that the overall area fraction of LFA-1-positive leukocytes was significantly larger in comparison with the area fraction positive for SST_2_-expressing macrophages independent of plaque phenotype. A large degree of variation was detected for fibrous cap atheroma segments in terms of the degree of positive LFA-1, reflecting the diverse inflammation status associated with this phenotype. The fibrous cap atheroma segments contained a significantly higher degree of inflammatory cells expressing LFA-1 compared with the fibrocalcific plaque segments and the pathological intimal thickening segments (Fig. 5a). For SST_2_, fibrous cap atheroma segments and fibrocalcific segments contained comparable area fraction levels of SST_2_-positive cells. Pathological intimal thickening segments contained a significantly lower amount of SST_2_-positive area (Fig. 5b).

### Radiotracer targeting of LFA-1 and SST_2_ in different plaque phenotypes assessed by SPECT

**Fig. 6:**
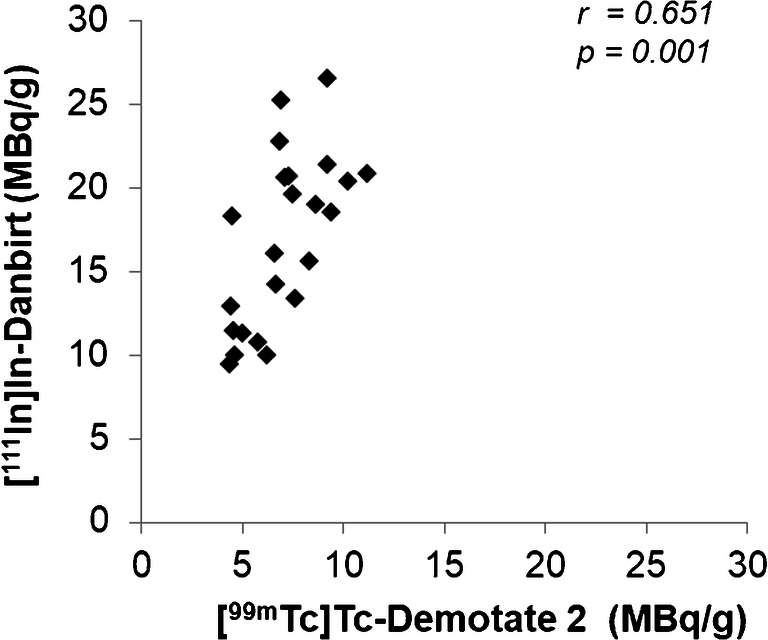
Positive correlation between [^99m^Tc]Tc-Demotate 2 and [^111^In]In-Danbirt and in terms of total uptake (MBq/g) per plaque segment

**Fig. 7:**
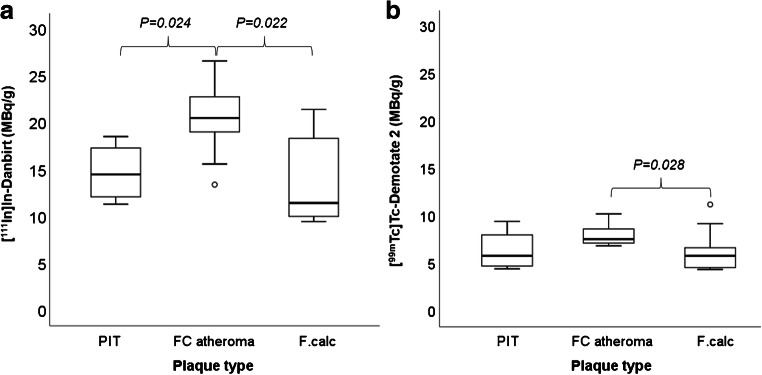
Box-plot of radiotracer uptake (MBq/g) for (**a**) [^111^In]In-Danbirt and (b) [^99m^Tc]Tc-Demotate 2 in each carotid plaque segment type classified according to plaque phenotypes. PIT, pathological intimal thickening; FC, fibrous cap atheroma; F.calc, fibrocalcific

High-resolution SPECT images portray the local specificity of the dual-radiotracer’s binding to inflamed regions in the plaques which also co-localises with positive immuno-stained regions of LFA-1 and SST_2_ (Fig. 3). An example of the focal high uptake of both radiotracers in the atheroma core and fibrous cap region of a fibrous cap atheroma plaque is illustrated in the SPECT images in Fig. 4 whereby regions of [^111^In]In-Danbirt uptake co-localised with [^99m^Tc]Tc-Demotate 2 uptake. The [^111^In]In-Danbirt uptake is significantly higher compared with the [^99m^Tc]Tc-Demotate 2 for all plaque samples analysed in this cohort (*p* = 0.001). There is a significant positive correlation in uptake between the radiotracers targeting the cells expressing LFA-1 and SST_2_ receptors (*r* = 0.651; *p* = 0.001) as illustrated in Fig. 6. Quantification of each radiotracer’s total uptake (MBq/g) in each 2-mm plaque segment with distinct plaque phenotype revealed that the highest uptake of [^111^In]In-Danbirt is correlated to plaque phenotype with fibrous cap atheroma (20.39 ± 3.97 MBq/g) (Fig. 7a). The uptake is significantly higher compared with both the pathological intimal thickening (14.70 ± 3.23 MBq/g) and fibrocalcific plaque (14.05 ± 4.89 MBq). For [^99m^Tc]Tc-Demotate 2 the mean uptake is significantly higher in the fibrous cap atheroma segments (7.98 ± 1.10 MBq/g) compared with the fibrocalcific segments (6.36 ± 2.39 MBq/g) (Fig. 7b). However, a significant difference was not reached between the fibrous cap atheroma and pathological intimal thickening (6.38 ± 2.23 MBq/g) for the [^99m^Tc]Tc-Demotate 2 uptake (*p* = 0.142).

**Fig. 8:**
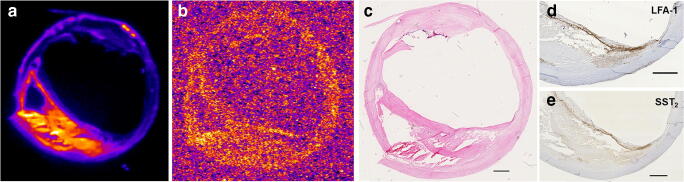
Uptake of dual-radiotracers in fibrous cap atheroma section; autoradiography images showing focal regions of high radiotracer binding for (**a**) [^111^In]In-Danbirt, (**b**) [^99m^Tc]Tc-Demotate 2, (**c**) haematoxylin and eosin stain demonstrating morphology of fibrous cap atheroma section and corresponding immuno-sections showing positive staining in the fibrous cap for (**d**) leukocytes (LFA-1) and (**e**) macrophages (SST_2_) (scale bar 1 mm)

Comparing the sections with and without excess (10^−6^ M) radiotracer revealed the substantial reduction in uptake in the CEA sections. The autoradiography analysis of [^111^In]In-Danbirt and [^99m^Tc]Tc-Demotate 2 uptake also revealed the focal regions of high radiotracer binding in the fibrous cap atheroma segments in line with the immuno-positive regions for LFA-1 and SST_2_ respectively (Fig. 8).

## Discussion

In this study, a dual-isotope acquisition SPECT imaging protocol was tested to assess radiotracer uptake in distinct plaque phenotypes pertaining to two inflammatory cell populations involved in atherosclerosis. We demonstrated the unique capability of the [^111^In]In-Danbirt to identify inflamed plaque phenotype using human carotid plaques acquired through endarterectomy procedures. The mean [^111^In]In-Danbirt and [^99m^Tc]Tc-Demotate 2 uptake was highest in fibrous cap atheroma plaque compared with pathological intimal thickening and fibrocalcific plaques. A comparison of the uptake for the two radiotracers revealed that [^111^In]In-Danbirt uptake was significantly higher compared with [^99m^Tc]Tc-Demotate 2 uptake for all plaque samples analysed in this cohort, independent of plaque phenotype. These findings were in line with the immunohistological findings, and detailed examination of the corresponding immunohistological sections revealed co-localisation with LFA-1- and SST_2_-expressing cells.

The atherosclerotic inflammatory activity is driven by multiple key leukocyte subsets including the monocyte-derived macrophages, lymphocytes and neutrophils [26, 27]. In this regard, it is important to detect the presence of all leukocyte subsets, encapsulating the total inflammation activity, as a means for early atherosclerotic disease detection and potential prevention of adverse cardiovascular events [28]. SPECT imaging showed focal uptake of [^111^In]In-Danbirt in the carotid plaque tissue which varied in accordance with the degree of inflammation that was heterogeneously distributed throughout the plaque as indicated by the co-localising areas of positive immuno-staining for LFA-1. The overall higher degree of [^111^In]In-Danbirt uptake compared with [^99m^Tc]Tc-Demotate 2 uptake for all plaque samples analysed in this cohort is indicative of the successful detection of all leukocytes which are playing a key role in the disease progression. In a clinical setting, PET scanning is the preferred methodology in atherosclerosis imaging in terms of nuclear medicine due to the superior spatial resolution and quantification capabilities compared with clinical SPECT. For example, Moto et al. recently demonstrated that Danbirt labelled with the PET radioisotope gallium-68 is highly stable in vitro in conditions that closely emulate those found during in vivo tracer delivery [9]. For clinical use, the equivalents to SPECT imaging with [^99m^Tc]Tc-Demotate 2 for targeting SST_2_-expressing proinflammatory macrophages in vivo are [^68^Ga]Ga-DOTA-TATE and [^64^Cu]Cu-DOTA-TATE which have both been shown to be potentially relevant tracers for plaque imaging with PET in cardiovascular disease patients.

From a pathological point of view, the atherosclerotic plaque composition is heterogeneous even within the same phenotype as identified for the [^111^In]In-Danbirt uptake in the fibrous cap atheroma segments. This is a consequence of the fact that inflammation levels vary considerably depending on the disease phase and inflammatory activity. Notably, this large variation in inflammation intensity was less pronounced in the [^99m^Tc]Tc-Demotate 2 uptake, likely as proinflammatory macrophages (SST_2_) represent a small subset of the inflammatory cells present in the plaque. While [^111^In]In-Danbirt imaging alone is very informative regarding atherosclerotic plaque phenotype detection, the ratio of [^99m^Tc]Tc-Demotate 2 to [^111^In]In-Danbirt advocates a potential role for dual-isotope acquisition in the assessment of plaque inflammation advancing the understanding towards the atherosclerotic disease development. For example, in the fibrous cap atheroma segments the ratio of [^99m^Tc]Tc-Demotate 2 to [^111^In]In-Danbirt is lower compared with the fibrocalcific segments. This implicates a higher level of involvement of the other leukocyte subsets with the proinflammatory macrophages in the fibrous cap atheroma phenotype compared with the more advanced fibrocalcific phenotype. In this regard, a dual-isotope acquisition protocol could provide added diagnostic benefit for the assessment of inflamed atherosclerotic vessels which could be useful for patient-specific therapeutic intervention.

Moreover, studies have reported the performance of [^111^In]In-Danbirt in vivo utilising the ApoE−/− mouse model. These studies provide evidence that the [^111^In]In-Danbirt is adequately taken up in local athero-prone regions, including the aortic arch. These atherosclerotic regions of uptake further co-localise with immunohistochemistry LFA-1-expressing cells [8]. Importantly, the myocardial uptake signal is sufficiently low with respect to the background levels so as to not interfere with the imaging quantification of the aorta’s inflammation [8].

Pathological studies have classified the fibrous cap atheroma phenotype as the ‘culprit’ part of the vessel [29–31]. Interestingly, in this study the intensity of radiotracer uptake correlated to plaque phenotype. The highest [^111^In]In-Danbirt uptake (MBq/g) correlates to inflamed fibrous cap atheroma plaque segments. This differentiates these highly inflamed plaque segments from fibrocalcific and pathological intimal thickening segments which are considerably less inflamed, and considered to have a lower risk of rupture. Similarly, the mean [^99m^Tc]Tc-Demotate 2 uptake was also highest in the fibrous cap atheroma segments and also significantly higher compared with the fibrocalcific phenotype in line with the immunohistochemistry. Notwithstanding, to make a distinction in specific atherosclerotic plaque phenotype and the identification of vulnerable plaque characteristics remains a challenge based on the current radiotracers used in the clinic [13, 32, 33]. For example, clinical studies have reported difficulty in differentiating symptomatic carotid vessels compared with the contralateral asymptomatic diseased side where there is a high degree of disease present in the contralateral carotid vessel [13]. Consequently, delineation of the culprit vulnerable plaque is not always possible. The typical morphological features of a vulnerable plaque have been determined from pathological studies and can be characterised using MRI scanning. These include the presence of a large lipid-rich necrotic core, an overlying thin fibrous cap layer and intraplaque haemorrhage [34–36]. However, morphological plaque data alone is also not enough for risk prediction of plaque rupture. In this regard, combining a measurement of the inflammation imaging [9] and the plaque morphology information from MRI may permit assessing the degree of inflammation activity coupled with vulnerable plaque characteristics in order to delineate high-risk vessels [37, 38].

There are inherent limitations associated with this study that must be taken into account. The clinical translational aspect of the dual-isotope acquisition imaging protocol utilised in this ex vivo setting needs to be assessed in vivo. Notably, the acquisition of SPECT/CT imaging involved using a HE-UHR-M collimator which resulted in a considerably higher in-plane resolution of 0.5 mm compared with the resolution capabilities of the clinical PET scanner which are approximately 10 times lower [33]. In a clinical setting, the opposite occurs whereby PET resolution outperforms SPECT. Thus the PET isotopes fluorine-18 and gallium-68 are more favourable in the clinical setting compared with indium-111 or technetium-99m, although there is no current option for dual-isotope imaging with the PET isotopes. Moreover, in order to incorporate a dual-isotope acquisition for SPECT imaging, [^99m^Tc]Tc-Demotate 2 was used simultaneously with [^111^In]In-Danbirt in this study. In the clinical setting, [^68^Ga]Ga-DOTA-TATE is utilised for PET imaging of SST_2_-expressing proinflammatory macrophages. It is important to take into account the application of different analogue radiotracers as they may lead to different quantitative results. Irrespective of the radiotracer applied, the fundamental principle of targeting the SST_2_ in atherosclerotic plaque tissue remains the same. Thus, the relative trend in uptake differences is expected to be comparable in the ex vivo and in vivo setting. In an in-vivo situation a larger degree of down scatter is to be expected in the ^99m^Tc window from ^111^In. This will subsequently increase the background signal for ^99m^Tc but should not deter from the image quality when scatter is corrected for. An increased amount of scattering from the tissue in an in vivo situation would also increase the background. The administration of two radionuclides will increase the radiation dose to the patient compared with the use of only one. Since both ^99m^Tc and ^111^In radionuclides radiate mainly low to medium energy gamma rays, and the absence of alpha and beta radiation, radiation issues are not expected when a diagnostic amount of these radionuclides are administered to the patient. In terms of the analysis, in an attempt to normalise the plaque segments for comparison, the radiotracer uptake was reported with respect to the weight of each plaque segment. In one plaque segment, classified with the pathological intimal thickening phenotype, a negligible degree of inflammation was present whereby positive area fraction of staining for LFA-1 and SST_2_ were 0.09% and 0.01% respectively on immunohistochemistry. As a consequence, it is likely that this ratio approach resulted in a false high uptake (MBq/g) for this one segment due to the extremely low uptake (MBq) in this segment and the low weight (g). Due to the small sample size in this phenotype group (*n* = 4), a significant difference therefore was not reached for [^99m^Tc]Tc-Demotate 2 uptake compared with the fibrous cap atheroma segments.

In conclusion, a dual-isotope acquisition SPECT scanning protocol was tested to assess the capability of [^111^In]In-Danbirt and [^99m^Tc]Tc-Demotate 2 to determine atherosclerotic plaque inflammation and phenotype. The novel [^111^In]In-Danbirt detects the presence of multiple leukocyte subsets and appears to be able to strongly discriminate plaque phenotype of fibrous cap atheroma from pathological intimal thickening and fibrocalcific plaque segments. This information provides a proof of concept basis for the [^111^In]In-Danbirt as a potential candidate for total inflammation imaging and plaque phenotype classification. Detecting the presence of all leukocytes in addition to leukocyte subset of proinflammatory macrophages may further enhance the pathological understanding of the inflammation involvement in atherosclerosis progression. Thus, further studies that evaluate the clinical benefit of utilising this radiotracer in vivo with clinical SPECT scanners are recommended.
